# Author Correction: Short tandem repeats delineate gene bodies across eukaryotes

**DOI:** 10.1038/s41467-025-56684-2

**Published:** 2025-02-04

**Authors:** William B. Reinar, Anders K. Krabberød, Vilde O. Lalun, Melinka A. Butenko, Kjetill S. Jakobsen

**Affiliations:** 1 Centre for Ecological and Evolutionary Synthesis, Department of Biosciences, University of Oslo, Oslo, Norway; 2 Section for Genetics and Evolutionary Biology, Department of Biosciences, University of Oslo, Oslo, Norway

**Keywords:** Comparative genomics, Genome evolution

Correction to: *Nature Communications* 10.1038/s41467-024-55276-w, published online 30 December 2024

In the version of the article initially published, in Fig. 1a, “Dikarya” was wrongly listed in the “Animal” section when it should have been part of the “Fungi” section. This has now been corrected in the HTML and PDF versions of the article.

