# Supplementary material for: Short tandem repeats delineate gene bodies across eukaryotes
Source: Nat Commun. 2024 Dec 30;15:10902. doi: 10.1038/s41467-024-55276-w (PMC11686069; doi:10.1038/s41467-024-55276-w)
Supplement: Supplementary file 1 — Supplementary Information [file 41467_2024_55276_MOESM1_ESM.pdf]

SUPPLEMENTARY INFORMATION

Supplementary Table 1. Tests of phylogenetic signal (Pagel's λ) in the mean gene-proximal repetitiveness scores of monomer and dimer motifs across 891 species with phylogenetic data available from TimeTree[1]. Pagel's λ was calculated using the phyloSignal[2] v.1.3.1 R package using 999 repetitions (reps = 999) with a TimeTree phylogeny and average gene-proximal repetitiveness scores for 891 species. All P-values falls beneath the Bonferroni adjusted P-value for multiple tests (0.005).

Motif	Pagel's λ	P-value
A	>0.99	≤ 0.001
T	>0.99	≤ 0.001
C	>0.99	≤ 0.001
G	>0.99	≤ 0.001
AT	>0.99	≤ 0.001
CG	>0.99	≤ 0.001
AC	>0.99	≤ 0.001
AG	>0.99	≤ 0.001
CT	>0.99	≤ 0.001
GT	>0.99	≤ 0.001

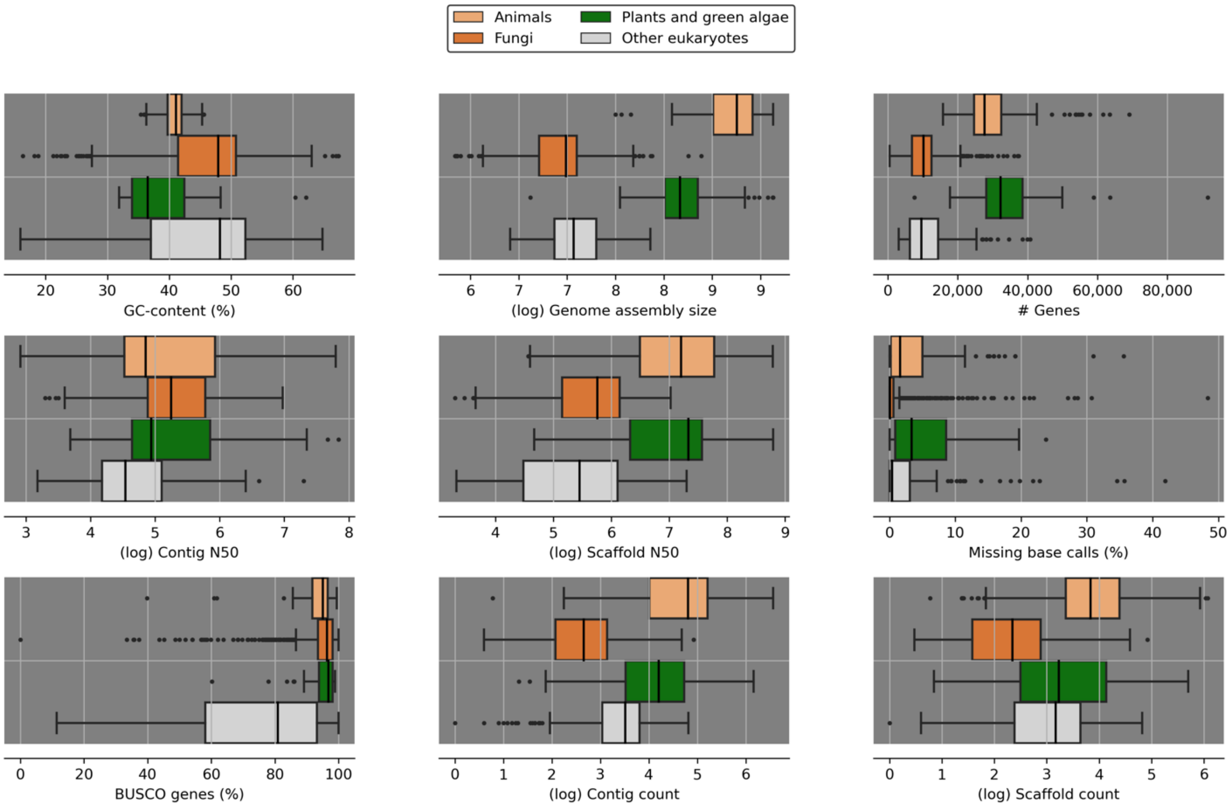

Supplementary Fig. 1. Genome architecture and genome assembly metrics of the surveyed species. The boxplots

show the variation in genome architecture metrics (genome size, GC-content, and gene count) and genome assembly metrics [contig/scaffold sizes/counts, missingness, and Benchmarking Universal Single-Copy Orthologs (BUSCO) genes] for the surveyed species. Lines within boxes indicate the median value, the box captures values within the 25th and 75th percentile, and the whiskers indicate the limits of the interquartile range multiplied with 1.5 – values outside whiskers are shown as points. ANOVA tests rejected the hypothesis of equal group means for all nine metrics [F-statistics: 40.6 (GC-content), 2972.1 (Genome assembly size), 844.6 (# Genes), 35.1 (Contig N50), 380.5 (Scaffold N50), 45.1 (Missing base calls), 176.3 (BUSCO genes), 456.0 (Contig count), 238.1 (Scaffold count), all P-values < 0.0001].

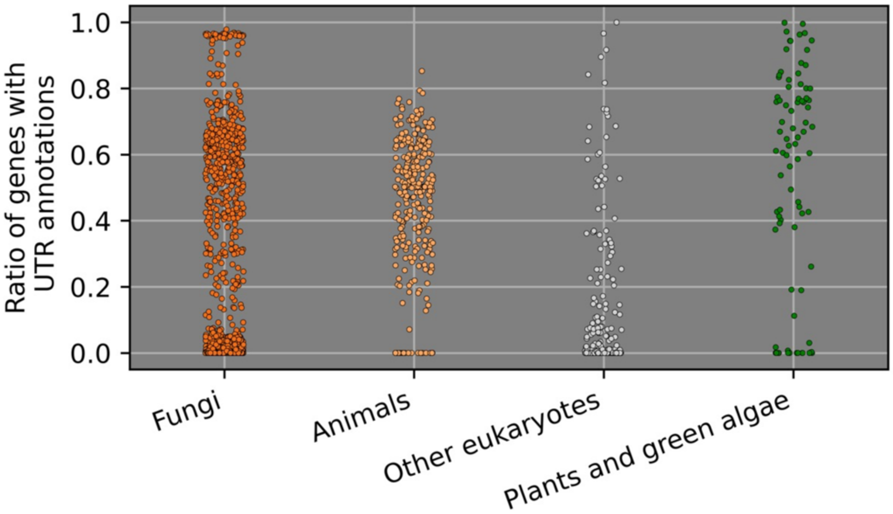

Supplementary Fig. 2. The ratio of genes with untranslated region (UTR) annotations (y-axis) per group (x-axis).

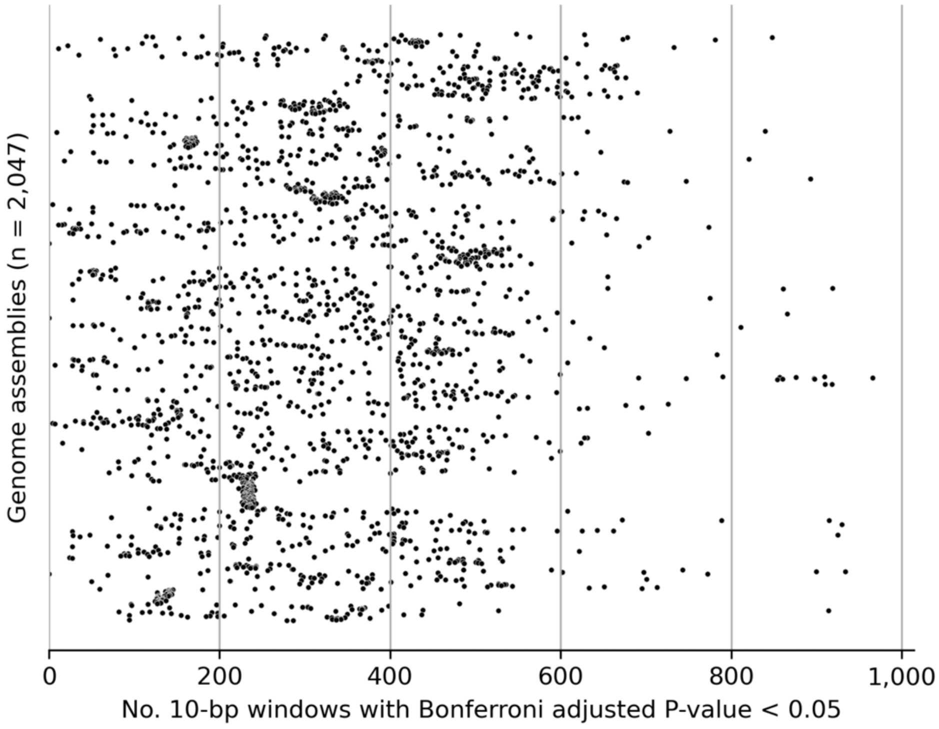

Supplementary Fig. 3. 10-bp windows with statistically significant shifts in repetitiveness. The number of 10-bp windows out of the 2,000 10-bp windows (x-axis) with a statistically significant difference in mean repetitiveness when compared to the region-specific mean is shown per genome assembly (y-axis). The null hypotheses of equal means were evaluated with two-sided T-tests where the degrees of freedom depended on the number of genes analyzed (*Methods*). The P-values were adjusted for the 4,094,000 tests using Bonferroni. All 2,047 genome assemblies had at least one window significant at the adjusted P-value threshold except for the genome assemblies *Leucoagaricus_sp_symc_cos_gca_001563735.ASM156373v1,* *Trypanosoma_rangeli_sc58_gca_000492115.T_rangeli_SC58v1*, and *Fomitiporia_mediterranea_mf3_22_gca_000271605.Fomme1.*

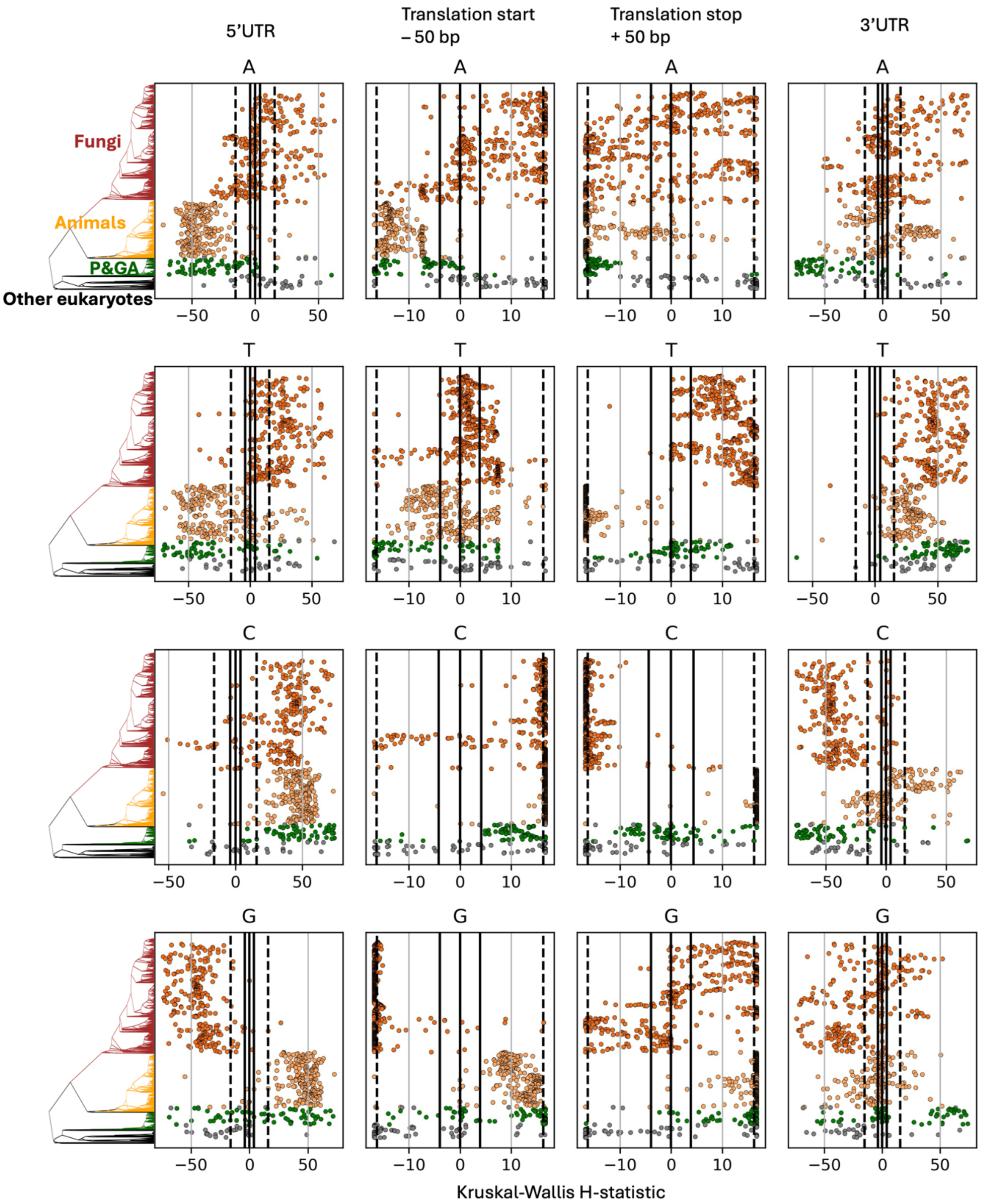

Supplementary Fig. 4. Kruskal-Wallis H-statistics per species for the repetitiveness of monomer motifs. The H-statistic reflect the difference in median sequence repetitiveness between the sequence upstream of 5'UTR annotations (up to 1,000 bp) and the 5'UTR sequences (leftmost panels) and between sequences downstream of 3'UTR annotations (up to 1,000 bp) and the 3'UTR sequences (rightmost panels). The middle panels show the region from the translation site to –50 bp upstream compared to the remaining upstream region (up to –1,000 bp) and the region from the translation stop site to +50 bp downstream compared to the remaining downstream region (up to +1,000 bp). Datapoint are sorted and colored according to the phylogenetic tree as drawn on the y-axis. Note that the H-statistic is represented with negative values when the region of interest had lower repetitiveness than its compared region. Solid lines indicate the lowest H-statistic reaching the nominal 0.05 P-value α-threshold and dashed lines indicate the lowest

H-statistic reaching the nominal P-value α-threshold when α were corrected for multiple testing. Source data are provided as a Source Data file.

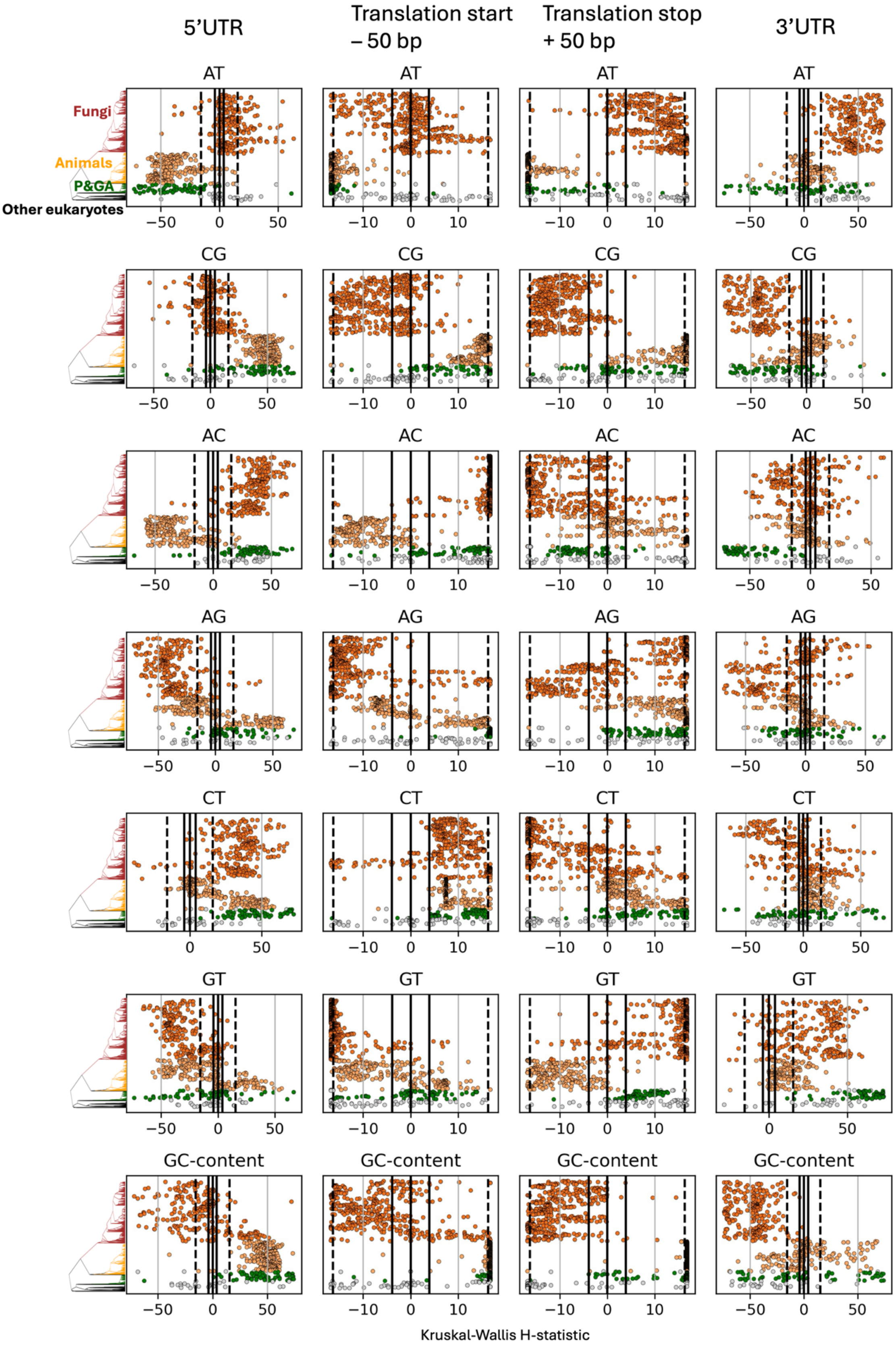

Supplementary Fig. 5. Kruskal-Wallis H-statistics per species for the repetitiveness of dimer motifs. Note that the H-statistic is represented with negative values when the region of interest had lower repetitiveness than its compared region. Solid lines indicate the lowest H-statistic reaching the nominal 0.05 P-value α-threshold and dashed lines indicate the lowest H-statistic reaching the nominal P-value α-threshold when α were corrected for multiple testing. Source data are provided as a Source Data file.

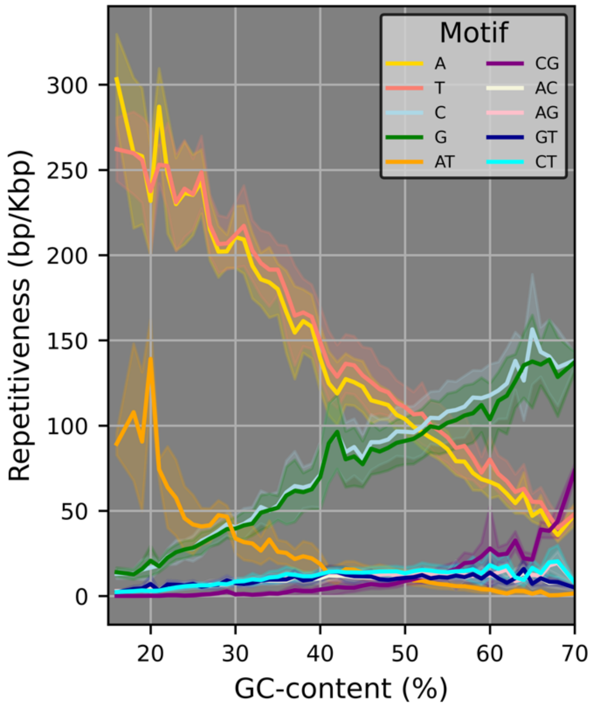

Supplementary Fig. 6. Repetitiveness as a function of genome-wide GC-content. Each line indicates the average repetitiveness ± 1 standard deviation (shaded areas) in 1,000 bp regions upstream and downstream of gene annotations (y-axis) as a function of GC-content (x-axis), per motif (see legend). N = 2,047 genome assemblies per motif. Source data are provided as a Source Data file.

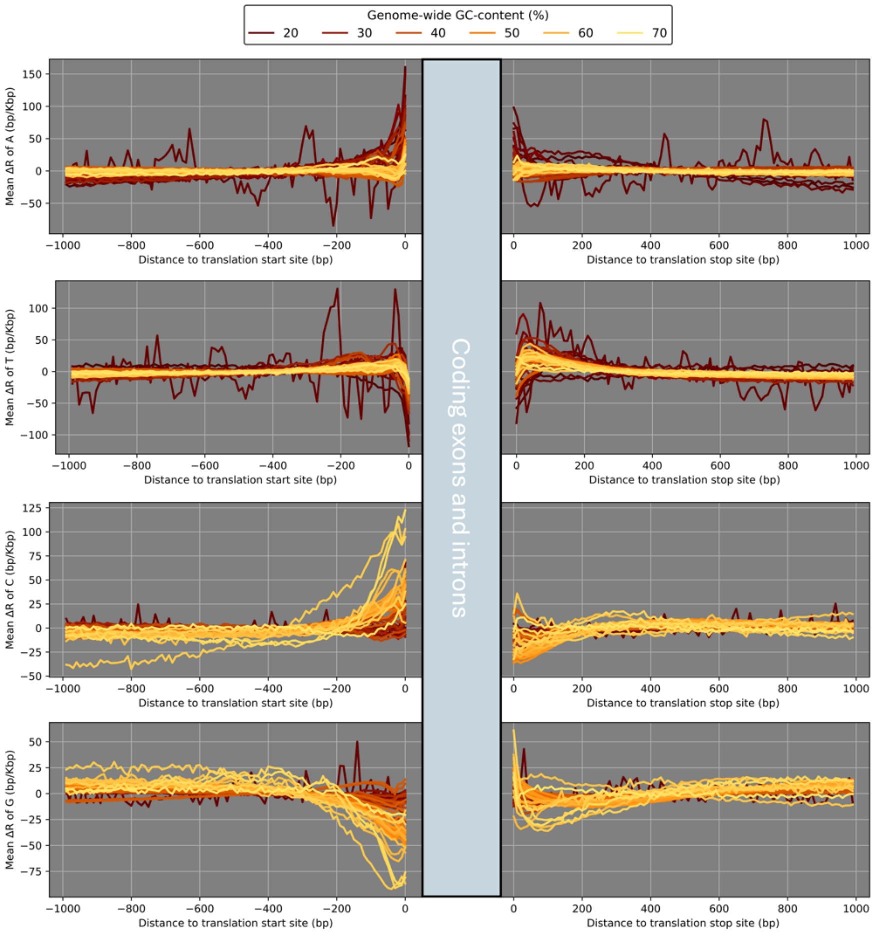

Supplementary Fig. 7. Shifts in monomer repetitiveness as a function of genome-wide GC-content. Mean ΔR_x-scores repetitiveness (y-axis) as a function of the distance to translation start sites (left panels) and translation stop sites (right panels), conditioned on the repeat motif (see y-axes). Source data are provided as a Source Data file.

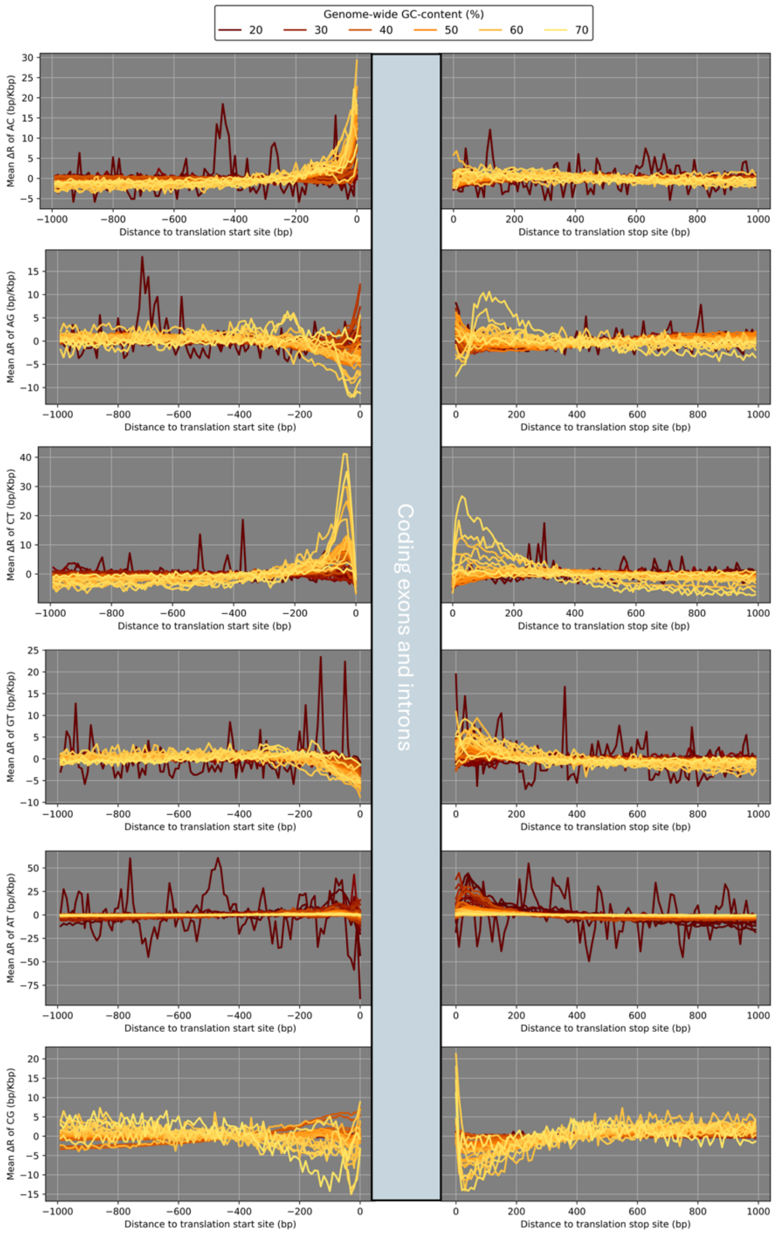

Supplementary Fig. 8. Shifts in dimer repetitiveness as a function of genome-wide GC-content. Mean ΔR_x-scores repetitiveness (y-axis) as a function of the distance to translation start sites (left panels) and translation stop sites (right panels), conditioned on the repeat motif (see y-axes). Source data are provided as a Source Data file.

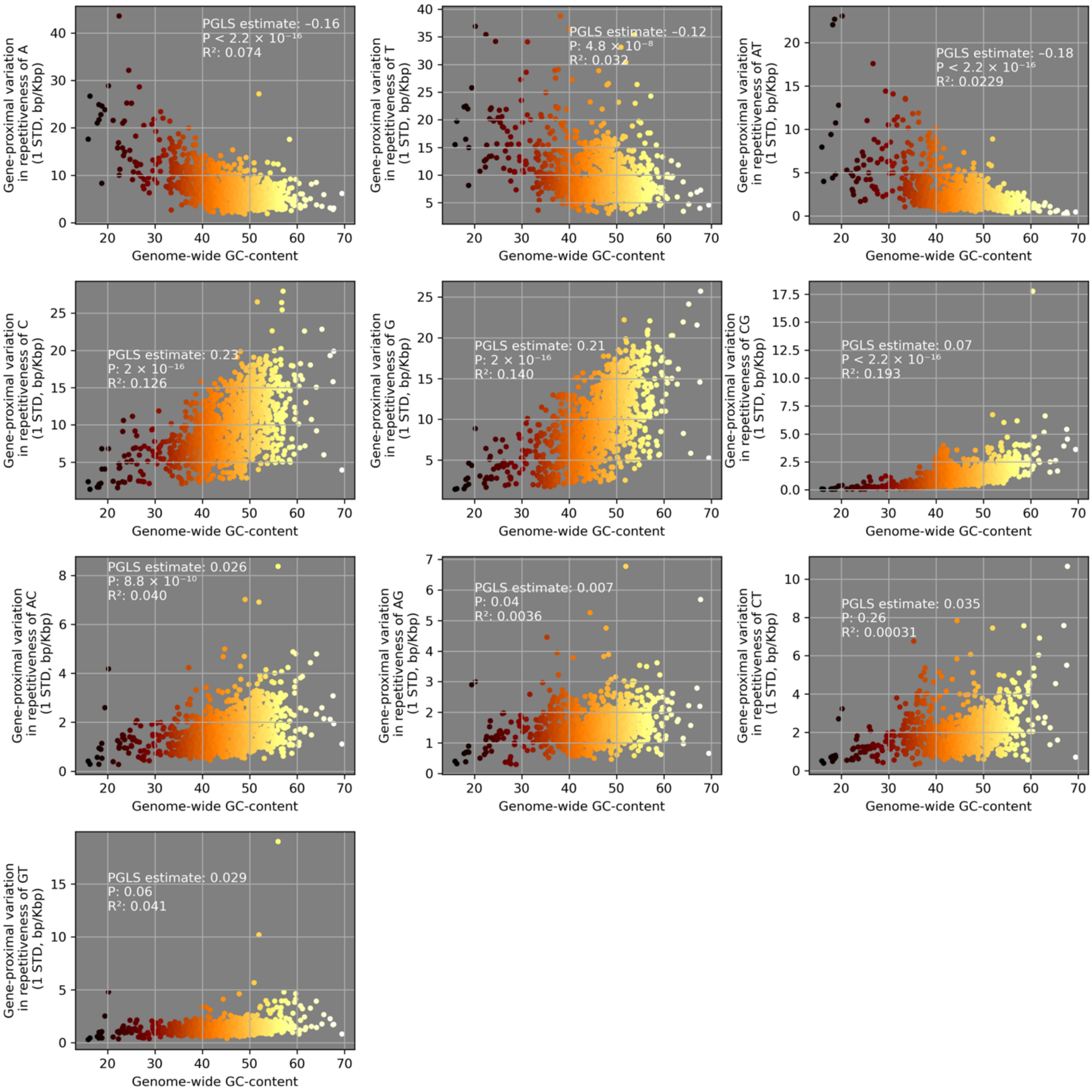

Supplementary Fig. 9. Variation in gene-proximal monomer and dimer repetitiveness in light of genome-wide GC-contents. Each datapoint shows the variation (one standard deviation; SD) in gene-proximal repetitiveness for one species as a function of its genome-wide GC-content (x-axis). Each panel shows data for one motif (A, T, AT, C, G, CG, AC, AG, CT, and GT). The text within the panels indicates the estimate (slope), P-value and R^2 value for each phylogenetic generalized least-squares (PGLS) model where the variation in the gene-proximal motif repetitiveness was modeled as a linear function of genome-wide GC content taking the dependence of datapoints (i.e., their phylogenetic relationship) into account. The PGLS model was used as implemented in the caper[3] R-package with maximum likelihood estimation of the lambda (branch length) parameter. Datapoints are colored according to the genome-wide GC-content. Adjusted P-value for multiple comparisons = 0.005. Source data are provided as a Source Data file.

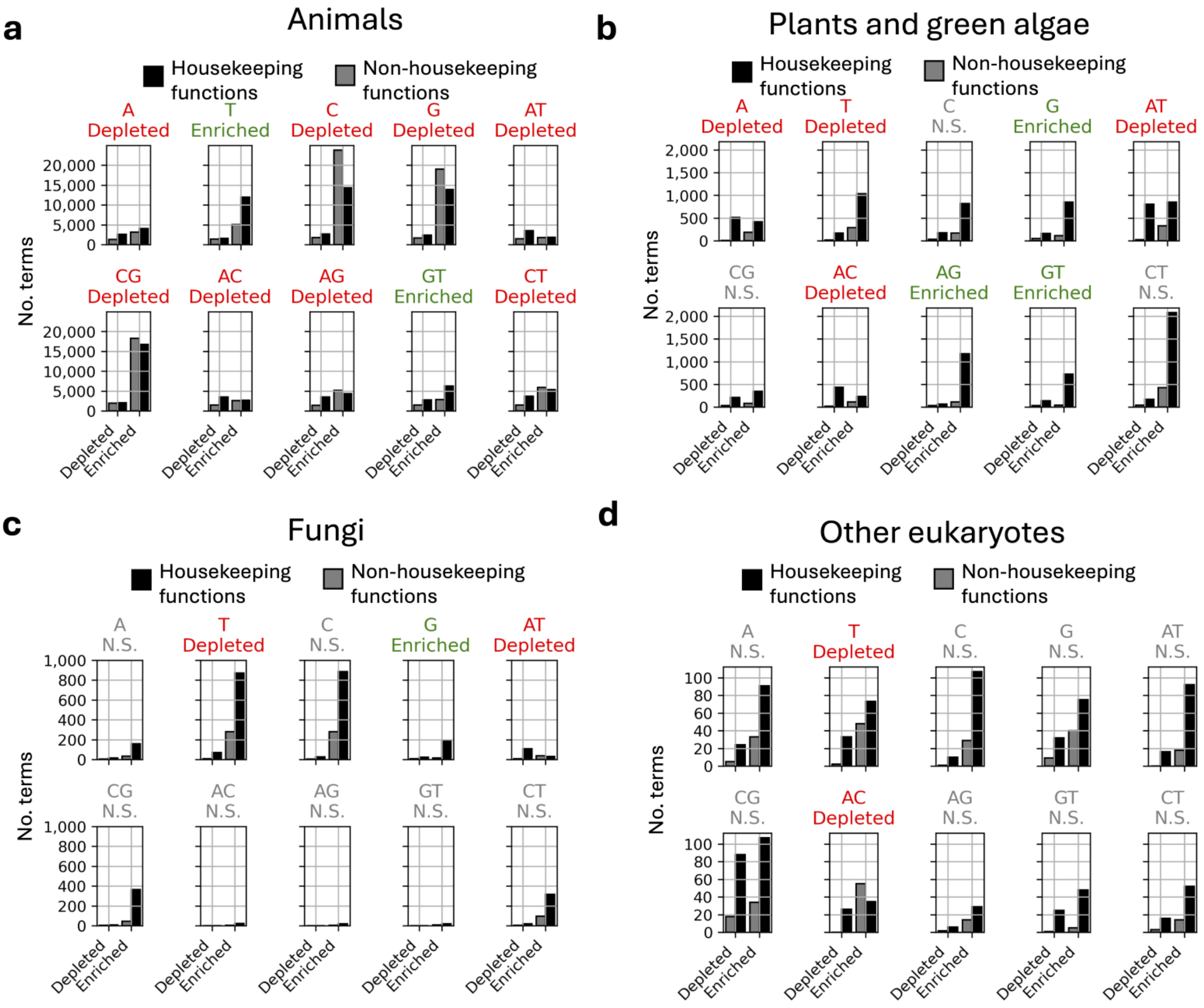

Supplementary Fig. 10. Enrichment and depletion of housekeeping functions in high-scoring gene lists in terms of variation in their gene-proximal repetitiveness. The bar plots indicate the number of GO terms (dark gray: non-housekeeping functions, black: housekeeping functions) that were statistically depleted or enriched (x-axes) in the gene lists per motif and per eukaryotic group (a: Animals, b: Plants and green algae, c: Fungi, d: Other eukaryotes). Two-sided Fisher's exact tests were used to assess the significance. N.S., non-significant. Source data are provided as a Source Data file.

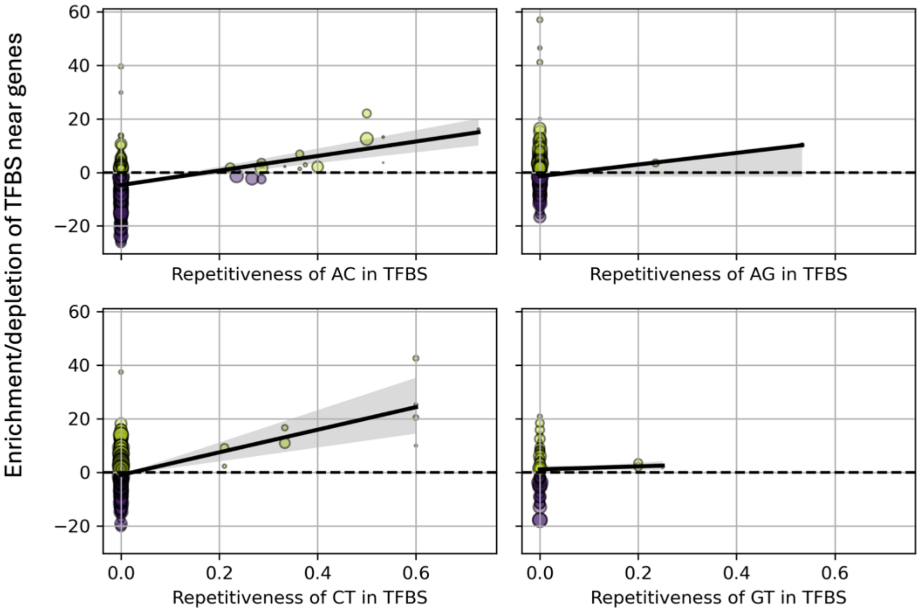

Supplementary Fig. 11. Enrichment/depletion scores of transcription factor binding sites (TFBS) in light of AC, AG, CT, and GT repetitiveness in the TFBS. The scatter plots indicate the depletion/enrichment scores of TFBS in gene-proximal regions of the 10% high-scoring genes in terms of variation in repetitiveness as a function of the repetitiveness of the TF binding site for motifs AC (top left), AG (top right), CT (bottom left), and GT (bottom right). Circle sizes are scaled with the number of genes associated with the TFBS. Yellow color indicates a statistically significant enrichment, and purple color indicates a statistically significant depletion. Linear fit, AC: $R^2 = 0.06$, P-value = 5.4×10^{-16}, AG: $R^2 = 0.006$, P-value = 1.2×10^{-2}, CT: $R^2 = 0.09$, P-value = 5.5×10^{-29}, GT: $R^2 = 0.001$, P-value = 0.74. Source data are provided as a Source Data file.

SUPPLEMENTARY REFERENCES

1. Kumar, S. et al. TimeTree 5: An Expanded Resource for Species Divergence Times. *Mol. Biol. Evol*. **39**, msac174. 2022.

2. Keck, F., Rimet, F., Bouchez, A. & Franc, A. phylosignal: an R package to measure, test, and explore the phylogenetic signal. *Ecol Evol.* **6**, 2773-2780 (2016).

3. Orme, D. et al. caper: Comparative Analysis of Phylogenetics and Evolution in R. R package version 1.0.3 (2023).
